# Effect of standing posture during whole body vibration training on muscle morphology and function in older adults: A randomised controlled trial

**DOI:** 10.1186/1471-2318-10-74

**Published:** 2010-10-15

**Authors:** Monica Mikhael, Rhonda Orr, Fleur Amsen, David Greene, Maria A Fiatarone Singh

**Affiliations:** 1 Exercise, Health and Performance Faculty Research Group, Faculty of Health Sciences, The University of Sydney, The University of Sydney, Sydney, NSW, 2006, Australia; 2 Vu University, De Boelelaan 1105, 1081 HV Amsterdam, Netherlands; 3 School of Exercise Science, Faculty of Health Sciences, Australian Catholic University, 25A Barker Road, Strathfield, NSW, 2135, Australia; 4 Faculty of Medicine, University of Sydney, Sydney, Australia; Hebrew SeniorLife and Jean Mayer USDA Human Nutrition Centre on Aging at Tufts University, 150 Harrison Ave Boston, Massachusetts 02111, USA

## Abstract

**Background:**

Whole body vibration (WBV) is a novel modality of exercise shown to improve musculoskeletal function. This study aims to examine the effects of standing posture during low magnitude WBV training on muscle function and muscle morphology in older adults.

**Methods:**

Nineteen men and women (50-80 years) were recruited to a three month randomised controlled trial and allocated to one of three groups: WBV with flexed knees (FK), WBV with locked knees (LK), or sham WBV with flexed knees (CON). Exposure was intermittent (1 min WBV:1 min rest) for 20 min, three times per week for 13 weeks. Measurements were taken at baseline and at three months. Primary outcomes included upper and lower body muscle function (strength, power and velocity). Secondary outcomes were muscle morphology, balance, habitual and maximal gait velocity, stair climb power, and chair stand performance.

**Results:**

Sixteen subjects completed the study. Relative (%) upper body contraction velocity improved significantly after WBV with FK compared to LK (FK 16.0%, LK -7.6%, CON 4.7, p = 0.01). Relative upper body strength (LK 15.1%, p = 0.02; FK 12.1%, p = 0.04; CON 4.7%) increased significantly following WBV compared to control. Absolute (p = 0.05) and relative (p = 0.03) lower leg strength significantly improved with both standing postures (LK 14.4%; FK 10.7%; CON 1.3%). Only the LK group differed significantly from CON in relative leg strength gains (p = 0.02). Potentially clinically meaningful but statistically non-significant improvements in lower leg muscle cross-sectional area (LK 3.7 cm^2^, FK 2.4 cm^2^, CON 2.2 cm^2 ^p = 0.13) were observed after WBV with LK compared to the other groups. No significant effects of WBV on any functional performance tests were observed.

**Conclusions:**

Our results suggest that WBV may improve muscle strength and contraction velocity in some muscle groups in older adults. However, hypothesised differential adaptation to standing posture (FK > LK) was observed only for upper body contraction velocity, making recommendations regarding this prescriptive element inconclusive. The efficacy, mechanism of action and long term feasibility of WBV for musculoskeletal health in older adults warrants continued investigation in robustly designed, sufficiently powered future studies.

**Trial Registration:**

ACTRN12609000353291.

## Background

Age-related loses of skeletal muscle mass (sarcopenia) [1,2] and altered neuromuscular activation manifest as changes in muscle function in older adults [3,4]. Muscle weakness, reduced muscle power and slower contraction velocity are amongst these functional changes [5-9] and are important and prevalent risk factors for falls, frailty, disability and loss of functional independence in the aged [9-12]. Sarcopenia is common, being reported to occur in 40% of people aged 80 years and older [13], highlighting the risk of disability and loss of independence in this cohort.

Presently, pharmacological treatment [14-19], and resistance exercise [20,21] are available to alleviate age-related muscular deterioration [22]. However pharmacological methods have been variably successful, often resulting in unwanted side-effects [22,23]. Resistance training has been shown to be an effective method to counteract sarcopenia [21]; though for some older individuals with very advanced frailty or certain diseases, robust resistance exercise may be unavailable or precluded [5,24]. Whole Body Vibration (WBV) exposure has therefore been proposed as a potentially safe, low-intensity alternative to current modalities to combat sarcopenia in exercise-intolerant, exercise-aversive or mobility-limited individuals, without the potential risks or behavioural barriers associated with high intensity exercise.

WBV uses high-frequency mechanical stimuli generated by a vibrating platform which are transmitted through the body [25]. The mechanical stimuli produced are thought to use neural pathways, stimulating muscle spindles, the sensory receptors located within the belly of the muscle. The 1a afferent signals are transmitted monosynaptically to activate alpha-motoneurons, initiating muscle fibre contractions [26,27]. The vibration stimulus is thought to result in a tonic vibration reflex, or tonic contraction of the muscle [28].

Improvements from WBV exercise have been reported in muscle function (strength [2,29-33], power [2,32], velocity [32]), balance [29,34-37], a reduction of muscle spasticity in those with cerebral palsy [38], and postural control in those with Parkinson's Disease [39]. However, many studies have included resistance training with non-uniform resistance protocols between WBV and non-WBV groups [25,26,40,41], leaving the mechanism of improvements unclear. Few studies [32,42] have examined the effect of WBV alone (without concomitant exercise on the platform), and some trials [1,42] have reported non-significant musculoskeletal adaptations. Furthermore, the ideal vibration dose, time course, frequency and posture to elicit an optimal response remain uncertain.

Although the posture adopted during WBV exercise has been observed to significantly influence the transmission of vibration to the skeleton [2,32,43], no studies have specifically investigated posture and its influence on adaptation to WBV. Vibration has been observed as best transmitted up the skeleton when standing with a straight, erect posture [44]. Using transcutaneous pins in the spine and femur, Rubin *et al *[44] reported a higher transmissibility of vibration through the skeleton when participants stood with locked knees, compared to a relaxed or flexed knee posture. Performing static exercise concomitant with flexed knee condition during WBV training has been shown to significantly increase activation of leg muscles [45,46]. Significant improvements in muscle strength and power after WBV exposure have been reported when subjects stood with knees slightly flexed [2,32]. Russo *et al *[32] found a significant increase in leg muscle power and velocity of contraction, and Rees *et al *[2] reported increases in both ankle plantar-flexion torque and power when comparing WBV to a control group. Together these results suggest that knee flexion may facilitate muscle improvements during WBV exposure.

However, to our knowledge, no studies have *directly *examined the effect of knee posture adopted during WBV on muscle adaptations. Robust, well-designed studies, particularly in older adults at risk of sarcopenia, are required in order to determine the most effective vibration prescription to elicit muscle adaptations. The literature suggests that a single WBV dose may be differently transmitted to muscle tissue dependent on knee position. Therefore the aim of this study was to determine the effect of knee position adopted during WBV on adaptation to the vibration stimulus in muscle in older adults, in order to refine the WBV prescription for these important outcomes. Specifically, we hypothesised that: (1) WBV exposure would improve all outcomes relative to Control and (2) WBV exposure with flexed knees would dampen skeletal vibration transmission by absorbing energy into muscles, thereby enhancing muscle activation, contractile activity and adaptations.

## Methods

### Study Design

The study was a three-month double blind, randomised placebo-controlled clinical trial to investigate the effect of knee position during WBV exposure on muscle function, muscle morphology and physical performance in older adults. Outcome measures were assessed at baseline and three months. The primary analytic strategy was complete case analysis, without regard to intervention adherence, and without imputation for missing data.

#### Randomisation

Participants were randomised to one of three groups following the completion of baseline assessments by an investigator independent of the study via a computerised program [47] using the method of randomly permuted blocks. Strata used included gender, age and use of osteoporosis medication, and randomisation was performed in blocks of six. Allocation concealment was complete, as participants were notified of their group assignment via a sealed, opaque envelope distributed after the completion of all baseline assessments.

### Participants

Participants were recruited by means of posters and articles in local medical, physiotherapy and dental practices, pharmacies, community businesses, Senior citizens, Bowling and Returned Soldiers' Leagues Clubs, senior websites, online forums, newsletters, newspapers, and via letterbox drops to houses. Recruitment occurred during March to June 2009.

Participant screening was conducted using a telephone questionnaire. Inclusion criteria were non-institutionalised adults aged 50 and older (women at least one year postmenopausal), no cognitive impairment, able to stand unaided for at least 20 minutes, perform a partial squat for 60 seconds, and be willing to participate in the study. If currently on medications for osteoporosis (e.g. bisphosphonates, Vitamin D, calcium), dosages had to be maintained for the duration of the study. Exclusion criteria included: contraindications to vibration exposure (pacemaker, current kidney or gall stones, acute lower back pain, blood clot or thrombosis within the last six months, fracture or joint replacement within the past 12 months, vibration-related injuries, amputation of lower extremities other than toes, Raynaud's disease), contraindications to strength testing, active malignancy or a terminal or rapidly progressive illness, and diseases related to bone metabolism other than osteoporosis, such as Paget's disease, end-stage renal failure, rheumatoid arthritis or multiple myeloma.

Written informed consent was obtained from all participants prior to enrolment. The study was approved by the University of Sydney Human Research Ethics Committee and registered under the Australia New Zealand Clinical Trials Registry (Number: ACTRN12609000353291).

### Intervention

#### Whole Body Vibration Exposure

Participants were assigned to one of three groups (two experimental intervention groups and one sham group) for a three-month period. The WBV dose and posture paradigms are described in Table 1. WBV exposure for all groups was intermittent (1 min vibration:1 min rest) for 20 min, 3 days per week for 3 months (13 weeks). The total number of exercise sessions was 39. WBV was conducted standing position on a synchronous vibration platform engineered by Australian Catholic University (2004). The motor speed controller was calibrated to vibrate at a frequency of 12 Hz. This frequency setting was achieved by attaching a spring-loaded potentiometer underneath the platform base and measuring the vibration rate. The amplitude of the vibration (1 mm peak to peak) was determined by the size of the cam fitted to the motor shaft. All participants stood on the vibration platform with their feet shoulder-width apart, hands by their sides, and wore standardised thick cotton socks to prevent any dampening that might result from footwear [48].

**Table 1:** Whole Body Vibration Groups and Protocols

Whole Body Vibration Group	Vibration	Posture on Platform	Vibration Dose		
			
			Frequency (Hz)	Peak-to-Peak Displacement (mm)	Magnitude (Peak Acceleration) (*g*)
**FK**	Active	Flexed Knees (20°flexion)	12	1	0.3
**LK**	Active	Locked knees	12	1	0.3
**CON**	Sham Control	Flexed knees (20°flexion)	12	0	0

FK: Flexed Knees Group

LK: Locked Knees Group

CON: Control Group

We recognise that a four group, fully-factorial design would have been optimal to test the main effects of vibration and knee position, and the interaction of vibration and knee position on study outcomes. However, due to the pilot nature of the study, we chose three groups that would allow us to separate the effects of attention, vibration and standing posture and investigate two primary questions: (1) What is the effect of WBV exposure added to a constant knee position/exposure volume (Control/Flexed (CON) vs. Vibration/Flexed (FK))? and (2) What is the effect of knee flexion on a constant WBV exposure volume (Vibration/Locked (LK) vs. FK)? Although examined, the comparison of CON to LK group was not of primary interest, as we recognised it would not be possible to isolate the effects of the two experimental condition changes (knee position *and *vibration exposure) from the control condition.

**Figure 1:**
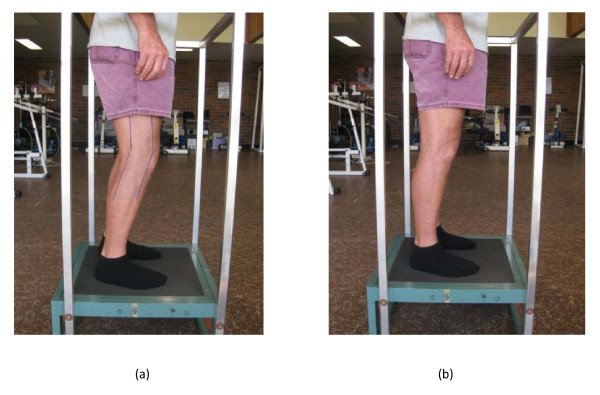
**Standing position on vibration platform**. (a) Flexed knees with marker, the standing position for the FK (flexed knee) and CON (Control) groups during the vibration or sham stimulus respectively, and (b) Locked knees, the standing position for the LK (locked kness) group during the vibration stimulus.

Each session was supervised by two trained research assistants in a University gymnasium. All participants were blinded as to which groups were hypothesised to be sham or active. Trainers by necessity were not blinded. The groups trained at separate times, to avoid unblinding and contamination. During CON training, the platform emitted a noise as the motor vibrated at a frequency of 12 Hz. However the amplitude was set to 0 mm, giving 0 *g *magnitude and providing a true sham control. During the FK conditions, a plastic sheet with the desired knee angle (20°) marked was taped to each participant's knee. During the LK condition, participants were instructed to lock knees but *not *to perform an isometric quadriceps contraction during the vibration exposure. The standing postures are depicted in Figure 1.

Attendance and completion of the exposure dose was monitored by the instructor at each session. Participants were permitted an additional four weeks to make up any missed sessions to reach their target 39 sessions.

### Outcomes

All outcomes were collected in double-blind fashion (both participants and assessors blinded). Baseline assessments were blinded (collected pre-randomisation) and measured by two assessors who also supervised the training sessions. Assessments at three months were measured by a different assessor blinded to the participants' group allocation and otherwise uninvolved in study procedures.

#### Primary Outcomes

##### Muscle Function (Strength, Power and Velocity)

Muscle function was assessed using Keiser pneumatic-resistance training equipment (Keiser Sports Health Equipment, Inc., Fresno, CA). Strength was measured using one repetition maximum (1RM) in two bilateral exercises: chest press and horizontal leg press. A 1RM was defined as the maximum load that can be lifted once while maintaining correct technique and reaching a full range of motion. Power and velocity were measured at 20, 30, 40, 50, 60, 70, 80, 90, and 100% of current 1RM and described in detail elsewhere [49]. The maximal explosive efforts were performed with 30-60 seconds rest in between [49].

#### Secondary Outcomes

##### Muscle Morphology

Total and regional (arms, legs and trunk) skeletal muscle mass and fat mass. Muscle-cross sectional area of mid-calf and mid-forearm were measured by Peripheral Quantitative Computed Tomography (pQCT) (Stratec XCT 2000; Medizintechnik, Pforzheim, Germany). The precision of repeat pQCT measurements was 0.7-1.4% (radius) and 0.8-2.9% (tibia) after repositioning in eight adults.

##### Physical Performance

Tests of physical performance included maximal (CV = 2.09%) and habitual (CV = 3.16%) gait speed over two meters, stair climb power, chair stand and six minute walk distance (CV = 5%) [50]. Balance, measured by balance index [49] was assessed on a computerised force platform (Chattecx Dynamic Balance System, Chattecx Corp, Chatanooga Group Inc, Hixson, TN; Software version 4.20). The mean of duplicate habitual gait velocity and maximum of duplicate maximal gait velocity and stair climb power measures were used. The remainder of the tests (balance, six minute walk, chair stand) were assessed only once at each time point.

### Covariates

#### Anthropometrics

Height (cm) was measured using a wall-mounted stadiometer (Holtain stadiometer; Holtain Limited, Crymmych Pembs, UK), and body mass (kg) was measured using an electronic scale (HW-100k, A&D Bench Scales, Bradford, MA). These were measured in triplicate and the mean value used to calculate body mass index (BMI) (kg/m^2^). The CV of triplicate measurement on the same day in the whole sample was 0.14% for height and 0.04% for body mass.

#### Demographics and Health Questionnaires

Demographic characteristics and self-reported medications and health conditions were assessed using a questionnaire. Habitual physical activity level was estimated by the Physical Activity Scale for the Elderly (PASE) [51]. All questions covered a seven-day period prior to completing the questionnaire, using results to compare physical activity reported between the two time points. A weekly questionnaire was conducted to monitor health status and possible adverse effects of vibration exposure. These were defined *a priori *by review of existing literature, and included questions probing details of illnesses, symptoms or injuries subjects may have experienced in the past week, changes in medications, visits to health care professionals, any changes in their physical, mental or emotional health and reasons for any missed exercise sessions.

### Statistical Analysis

Statistical analyses were performed using SPSS for Windows (Version 17.0). Data distributions were inspected visually and statistically for normality. Normally distributed data were reported as mean ± standard deviation (SD). Non-normally distributed data were normalised via log transformation, and if not possible, non-parametric statistics were used for these variables. Post hoc power analysis was calculated using the statistical program G Power (version 3.1.0) [52].

The primary analytic strategy compared the differences in primary and secondary outcomes between the locked knees, unlocked knees and sham WBV groups using all available data regardless of intervention compliance level. Analysis of covariance (ANCOVA) models of absolute change scores were constructed to compare the groups using the change score as the dependent variable and the baseline score as a covariate. Additional covariates considered for inclusion were characteristics that were different between the groups at baseline and related to the variable of interest (potential confounders). For muscle performance and body composition outcomes the month of each subject's baseline assessment was used as an additional covariate to control for the natural fluctuation of these parameters with changing seasons [53]. Fisher's post hoc Least Significant Difference (LSD) *t *tests were used for all pairwise comparisons whenever the f ratio in ANCOVA models was significant (*P *≤ 0.05), to ascertain which groups were different from each other. Hedge's bias-corrected relative effect sizes (ES) and 95% confidence intervals were calculated for selected outcomes by COE's calculator [54] as:

Change in Treatment Group - Change in Control Group/pooled baseline SD A p-value of ≤0.05 and/or 95% CI exclusive of zero were accepted as statistically significant. Cohen's definition of effect size was used (Negligible = 0.2; Low = 0.2 - 0.49; Moderate = 0.5 - 0.79; Large = 0.8+) [55].

## Results

### Recruitment

**Figure 2:**
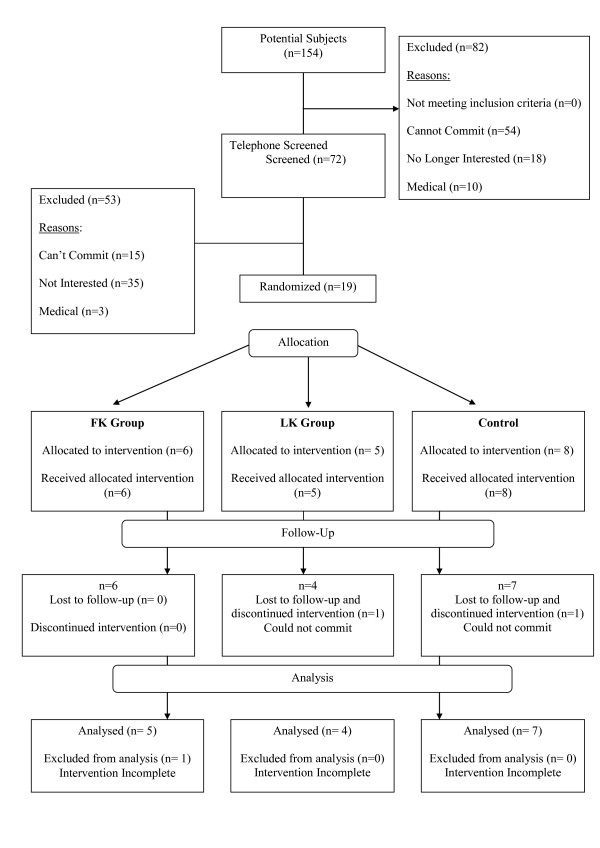
**Participant flow through the trial**.

From 154 persons assessed for eligibility, 19 were recruited and enrolled into the study (Figure 2). Two participants withdrew from the study (one after 14 weeks and the other following three sessions of exposure) due to personal reasons (LK = 1, CON = 1). One participant had not completed three months of exposure by the time of this analysis (FK = 1) and was not included in this report.

### Participant Characteristics

The mean age of participants was 64.4 ± 8.1 (range 50-80) years. Participants were generally healthy but overweight with an average of 2.2 co-morbidities, most commonly osteoarthritis (63%), hypertension (47%) and lower back pain (47%) (Table 2). At baseline, no significant or clinically important differences were found between groups for any characteristics (Tables 2 and 3).

**Table 2:** Baseline Participant Characteristics

Variable	Total (n = 19)	FK (n = 6)	LK (n = 5)	CON (n = 8)	p-value*
**Age (years)**	64.4 ± 8.1	63.3 ± 7.6	69.0 ± 6.9	62.3 ± 8.8	0.34
	8/11	4/2	3/2	4/4	
**Gender M/F, (%Female)**					0.47
	(58%)	(69%)	(60%)	(50%)	
**Height (cm)**	166.7 ± 9.5	166.7 ± 9.1	164.1 ± 6.1	168.4 ± 12.0	0.76
**Body Mass (kg)**	79.5 ± 15.8	72.9 ± 10.4	81.1 ± 9.7	83.4 ± 121.2	0.48
**Body Mass Index (kg/m**^**2**^**)**^**a**^	28.5 ± 4.3	26.2 ± 2.8	30.0 ± 2.3	29.2 ± 5.7	0.30
**Co morbidities/conditions, *n *(%)**					
**- Osteoarthritis**	12 (63.2)	4	3	5	0.97
**- Hypertension**	9 (47.4)	1	3	5	0.19
**- Chronic Lower Back Pain**	9 (47.4)	2	2	5	0.52
**- Osteoporosis**	5 (26.3)	1	1	3	0.64
**Total Co morbidities (#)**	2.2 ± 1.2	1.3 ± 1.2	2.6 ± 1.1	2.5 ± 1.1	0.13
**Total Medications/day (#)**	4 ± 3.9	4.5 ± 4.1	3 ± 1.2	4.4 ± 5.3	0.81
**Habitual Physical Activity Level**^**b**^	146.1 ± 44.1	137.3 ± 60.0	160.0 ± 49.9	145.8 ± 30.0	0.75
**Skeletal Muscle Cross Sectional Area (mm**^**2**^**)**^**c**^					
**- Mid-Calf**	67.8 ± 15.1	59.5 ± 12.9	72.6 ± 12.1	71.4 ± 17.6	0.27
**- Mid-Forearm**	33.5 ± 11.0	30.5 ± 11.3	35.0 ± 11.1	35.1 ± 11.8	0.728

All data presented as mean ± SD

Continuous variables analysed by ANOVA for normally distributed data

Categorical variables analysed by Chi square test

FK: Whole Body Vibration Flexed Knees Group

LK: Whole Body Vibration Locked Knees Group

CON: Control Group

^a ^Body mass index: an indicator of body fat calculated by weight (kg)/height^2 ^(m). Normal values range from 18.5 - 24.9 kg/m^2^.

Values ≥25 kg/m^2 ^are considered overweight, and ≥30 kg/m^2 ^are considered obese.

^b ^The Physical Activity Scale for the Elderly (PASE) was used to monitor habitual physical activity over the preceding seven days. A higher score reflects more physical activity [51]

^**c **^measured by Peripheral Quantitative Computed Tomography ( pQCT)

**Table 3:** Baseline Participant Functional Performance

		FK	LK	CON	
Variable	Total (n = 19)				p-value
		(n = 6)	(n = 5)	(n = 8)	
**Maximal Strength (N)**^**a **^-					
**- Leg Press**	1339.9 ± 416.1	1308.0 ± 579.2	1252.4 ± 289.6	1418.5 ± 380.0	0.78
**- Chest press**	327.1 ± 149.8	313.0 ± 190.9	293.0 ± 134.9	354.8 ± 137.8	0.79
**Relative Strength (kg/kg)**^**b -**^					
**- Leg Press**	8.9 ± 1.6	8.9 ± 2.1	8.4 ± 1.4	9.3 ± 1.5	0.38
**Peak Power (W) -**					
**- Leg Press**	652.3 ± 317.4	635.7 ± 438.3	637.6 ± 267.8	673.9 ± 282.6	0.97
**- Chest Press**	210.8 ± 108.4	191.3 ± 124.3	210.3 ± 69.4	225.6 ± 121.8	0.86
**Peak Velocity (cm/s) -**					
**- Leg Press**	94.5 ± 19.3	86.8 ± 21.8	89.3 ± 1.0	103.4 ± 20.1	0.23
**- Chest Press**	126.5 ± 32.0	115.1 ± 30.7	137.9 ± 16.8	129.4 ± 38.7	0.54
**Balance Index**^**c**^	98.8 ± 25.2	98.0 ± 20.0	106.348 ± 30.2	94.8 ± 27.8	0.74
**5 Chair rise time (s)**	10.1 ± 1.7	10.1 ± 1.8	10.1 ± 1.2	10.1 ± 2.0	1.0
**Stair Climb Power (W)**^**d**^	461.8 ± 195.9	420.2 ± 213.1	415.3 ± 92.4	530.6 ± 236.9	0.52
**Habitual Gait Velocity (m/s)**^**e**^	1.4 ± 0.2	1.3 ± 0.2	1.4 ± 0.2	1.3 ± 0.2	0.80
**Maximal Gait Velocity (m/s)**^**e**^	2.2 ± 0.4	2.2 ± 0.4	2.1 ± 0.3	2.3 ± 0.4	0.58
**Six Minute Walk (m)**	619.4 ± 77.0	612.5 ± 110.0	595.4 ± 50.3	639.5 ± 65.1	0.61

All data presented as mean ± SD

Continuous variables analysed by ANOVA for normally distributed data

FK: Whole Body Vibration Flexed Knees Group

LK: Whole Body Vibration Locked Knees Group

CON: Control Group

^a ^Maximal Strength was measured via 1 repetition maximum (1RM) testing in Newtons

^b ^Relative Strength (kg/kg): (leg press strength (kg)/leg Fat Free Mass (kg)) measured by Dual X-Ray absorptiometry (DXA)

^c ^Balance Index : (Sum of 12 sway measures + (180 - sum of 6 time measures) [49]

^d^Stair Climb power (W): (Body Mass (kg) × vertical height of the staircase (m)) × 9.8/time (s)

^e ^Gait velocity (average of 2 trials for habitual gait; maximal of 2 trials for maximal gait) measured over 2 m using an Ultratimer

### Compliance/Adverse Effects

No adverse events attributable to WBV exposure were reported. Compliance (number of vibration sessions completed divided by the 39 possible sessions available to each participant) for those completing the three months of WBV exposure was 98.8% with participants taking, on average, 42 days over 13-17 weeks to complete the 39 planned sessions.

### Outcomes

Participants' baseline body composition, muscle function, and physical performance are presented in Tables 2 and 3. Between group comparisons of change scores are presented in Table 4.

**Table 4:** Between Group Changes From Baseline to 3 Months

Variable	FK	LK	CON		
				p value	Power
	(n = 5)	(n = 4)	(n = 7)		
***Muscle Function and Mass***					

**Maximal Strength (N)**^**a **^-					
**- Leg Press**	93.03 (-4.3, 190.37)	202.32 (85.05, 319.59)	9.59 (-75.11, 94.30)	0.05*	0.59
**- Chest press**	-2.46 (-50.38, 45.50)	-3.72 (-98.91, 61.46)	-12.51 (-56.40, 31.38)	0.93	0.06
**Peak Power (W)**^**b **^-					
**- Leg Press**	41.19 (-23.99, 106.37)	95.73 (22.12, 169.35)	119.19 (65.74, 172.63)	0.18	0.29
**- Chest Press**	11.34 (-13.02, 35.70)	-0.09 (-47.04,47.02)	3.08 (-26.43, 32.58)	0.81	0.07
**Peak Velocity (cm/s)**^**b**^-					
**- Leg Press**	-10.36 (-140.71, 119.99)	43.02 (-104.10, 190.15)	59.53 (-48.98, 168.05)	0.68	0.10
**- Chest Press**	15.48 (3.59, 27.37)	-3.12 (-24.81, 18.58)	0.33 (-13.42, 14.08)	0.15	0.34
**Skeletal Muscle Cross - Sectional Area measured at 66% site (cm**^**2**^**)**^**b,c**^					
**- Mid-Calf**	2.43 (-2.02, 6.94)	3.65 (-1.91, 9.20)	-2.2 (-6.043, 1.65)	0.13	0.39
**- Mid-Forearm**	-0.24 (-1.52, 1.04)	-0.48 (-2.18, 1.23)	0.03 (-1.16, 1.22)	0.87	0.07
***Functional Performance***					
**Balance Index **^**d**^	0.73 (-10.83, 12.28)	-7.19 (-20.42, 6.04)	-11.35 (-22.24, -0.47)	0.28	0.24
**5 Chair Rise Time (s)**	-0.65 (-2.07, 0.78)	-1.29 (-2.87, 0.30)	-1.29 (-2.59, 0.01)	0.73	0.09
**Stair Climb Power (W)**^**e**^	84.10 (-28.02, 196.30)	63.68 (-55.17, 182.54)	42.75 (-63.86, 149.35)	0.86	0.07
**Habitual Gait Velocity (m/s)**^**f**^	0.40 (-0.02, 0.81)	0.04 (-0.04, 0.50)	0.07 (-0.30, 0.45)	0.38	0.19
**Maximal Gait Velocity (m/s)**^**f**^	0.09 (-0.11, 0.30)	0.01 (-0.22, 0.24)	0.09 (-0.1, 0.28)	0.81	0.75

**Six Minute Walk Distance (m)**	36.15 (3.18, 69.12)	22.92 (-14.27, 60.11)	31.58 (1.01, 62.15)	0.84	0.07

Data presented as Estimated Marginal Means, adjusted mean difference (95% Confidence Intervals) after Analysis of Covariance (ANCOVA) testing using baseline values as the covariate

All data presented as mean ± SD

WBV FK: Whole Body Vibration Flexed Knees Group

WBV LK: Whole Body Vibration Locked Knees Group

CON: Control Group

* Indicates a significant difference between the three groups (*p *≤ 0.05)

^a ^Maximal strength was measured via 1 repetition maximum (1RM) testing in Newtons

^b ^Estimated Marginal Means after Analysis of Covariance (ANCOVA) testing using baseline values and month of baseline pQCT scan as covariates

^c ^Measured by Peripheral Quantitative Computed Tomography (pQCT)

^d ^Balance index : (Sum of 12 sway measures + (180 - sum of 6 time measures) [49]

^e ^Stair climb power (W): Body Mass (kg) × vertical height of the staircase (m) × 9.8/time (s)

^f ^Gait velocity (average of 2 trials for habitual gait; maximal of 2 trials for maximal gait) measured over 2 m using an Ultratimer

#### Primary Outcomes

**Figure 3:**
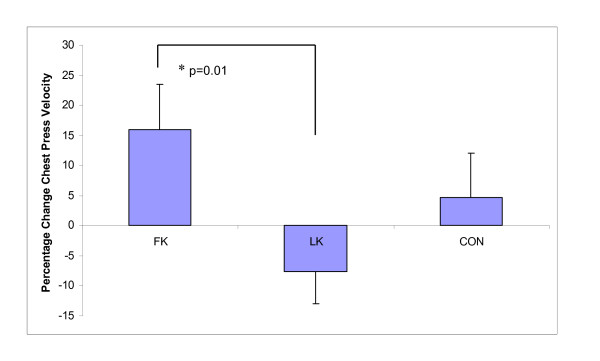
**Percentage change in chest press peak velocity following whole body vibration training**. Data presented as Estimated Marginal Means, adjusted mean difference ± standard deviation after analysis of covariance (ANCOVA) testing using month of baseline assessment and baseline values as the covariates. FK = flexed knees group, LK = locked knees group, CON = control group * significant between group change.

**Figure 4:**
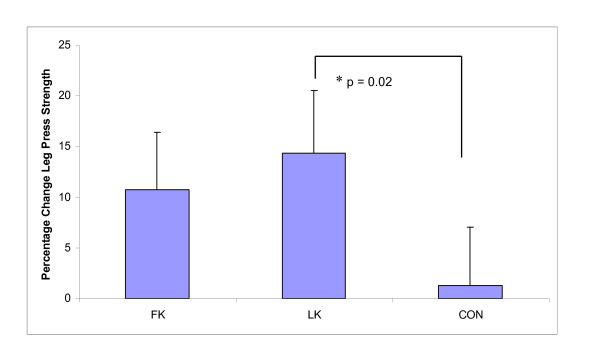
**Percentage change in leg press strength following whole body vibration training**. Data presented as Estimated Marginal Means, adjusted mean difference ± standard deviation after analysis of covariance (ANCOVA) testing using month of baseline assessment and baseline values as the covariates. FK = flexed knees group, LK = locked knees group, CON = control group * significant between group change.

**Figure 5:**
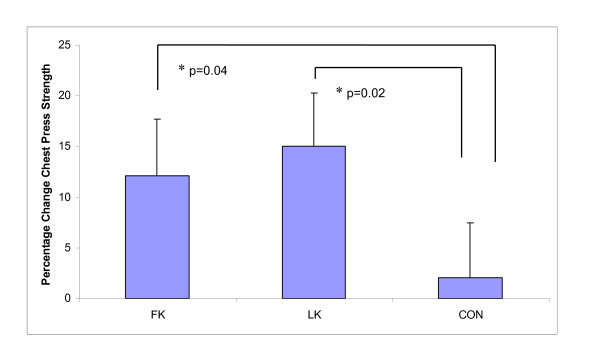
**Percentage change in chest press strength following whole body vibration training**. Data presented as Estimated Marginal Means, adjusted mean difference ± standard deviation after analysis of covariance (ANCOVA) testing using month of baseline assessment and baseline values as the covariates. FK = flexed knees group, LK = locked knees group, CON = control group * significant between group change.

As hypothesised, relative (%) upper body (chest press) peak contraction velocity significantly improved after WBV exposure in the FK group compared to LK (CON 4.7%; FK 16.0% vs. LK - 7.6%, p = 0.01) (Figure 3), and similar trends were observed for changes in absolute upper body velocity (Table 4). This was supported by a large effect size (ES) (ES = 0.82, 95% Confidence Intervals (CI) (-0.87, 2.52)). Relative (p = 0.03) and absolute (p = 0.05) lower leg (leg press) strength (Figure 4) increased significantly with both standing postures during WBV exposure, (LK 14.4%; FK 10.7%; CON 1.3%; ES 0.12, 95% CI (-1.15, 1.39)), however, unexpectedly more so with LK than FK. Furthermore, only the LK group was significantly different from CON in relative strength gains (p = 0.02). Similarly, relative upper body strength improved significantly following WBV compared to the control group (LK 15.1%, p = 0.02; FK 12.1%, p = 0.04; CON 4.7%; ES 0.05, 95% CI (-1.34, 1.43)) (Figure 5), however, contrary to our hypothesis, greater improvements were observed with LK than with FK. No significant changes were observed in peak muscle power in any group (Table 4).

#### Secondary Outcomes

Potentially clinically meaningful but statistically non-significant improvements in lower leg muscle cross-sectional area (LK 3.7 cm^2^, FK 2.4 cm^2^, CON 2.2 cm^2 ^p = 0.13; ES 0.28, 95% CI (-0.91, 1.48)) were observed after WBV with LK compared to FK or CON exposures. There were no other significant or clinically meaningful changes observed in physical performance over the three months (Table 4).

## Discussion

This is the first randomised controlled trial (RCT), to our knowledge, comparing different standing postures during WBV exposure in older adults. As hypothesised, three months of WBV exposure significantly improved absolute and relative lower body muscle strength and relative upper body strength and peak contraction velocity. The FK position was significantly better than LK for upper body muscle velocity improvement as hypothesised, but had no influence on muscle strength changes. A large calculated ES value for upper body contraction velocity adds support to the robustness of our findings for this outcome. This study is also to our knowledge, the first to report significant increases in upper body strength and contraction velocities after WBV. As expected, due to the pilot nature of this work (n = 16), type II errors likely contributed to the lack of significance for many of the other outcomes. The remainder of ESs were negligible to low, and all of the CIs included zero. Post-hoc power calculations of lower limb (leg press) strength indicated that a total of 78 subjects would be needed to demonstrate significance.

Chest press contraction velocity improved after WBV exposure, with an increase of 15.2% observed when standing in the FK position. Changes in contraction velocity may be the primary mechanism by which to improve peak power in older adults [56]. Because muscle power declines faster in older adults than strength does [57] and is more closely related to physical performance, functional independence and mobility than muscle strength [58,59] this finding may be clinically relevant. Muscle power has also been observed to improve with low, moderate and high load, high velocity power training [56]. Low load power training is most similar to the low-loading conditions during WBV exposure, although the frequency and number of contractions induced with the WBV would be far greater. Thus, WBV may provide an alternative exercise modality for those who cannot undertake power training. This, however, remains to be shown in future studies directly comparing the physiological and clinical benefits of power training vs. WBV, as no changes in muscle power itself were observed in this study. It is likely that greater improvements in muscle strength and/or velocity would have been necessary to improve power output itself [60].

### Muscle Strength

We observed significant differences in strength between WBV and CON participants after three months, as hypothesised. Despite statistical significance of the increase in upper and lower body strength, the increases are smaller than those typically observed following resistance training in older adults [61]. Thus, the clinical relevance and long-term benefits of strength changes associated with WBV remain to be demonstrated. Although we anticipated more robust changes in lower body muscle function than upper body changes, due to dissipation of vibration transmission over the length of the body, this was true for muscle strength, but not contraction velocity. With no previous literature available for comparison, these findings require further investigation through more robust studies, testing specifically for the influence of knee position during WBV exposure on upper and lower body muscle strength, power and contraction velocity.

### Effect of Standing Posture on Muscle Adaptations

The greater increase in upper body muscle velocity in the FK group over LK vs. CON supports our hypothesis that standing with flexed knees during WBV exposure facilitates adaptations in muscle. A similar vibration protocol was applied to investigate the transmissibility of vibration through to different areas of the skeleton when subjects adopted various knee positions [44]. The findings suggest that a flexed knee position may dampen vibrations to the skeleton. Similarly, our hypothesis proposed that this damping could actually facilitate the vibration adaptation muscle, resulting in better muscle function or morphology gains. Our results, however, provide inconsistent support for this hypothesis. Lower limb (leg press) strength improved significantly in both absolute and relative terms after three months of WBV exposure, however a greater increase was observed in the LK group than the FK group.

There are several explanations for these unexpected findings. Although all training was fully supervised and specific directions for standing position was given by the instructor, subjects could have unconsciously performed an isometric contraction of the quadriceps intermittently to stabilise the LK position during the WBV exposure. This may have inadvertently made the LK position more effective for muscle than the FK position, not due to dampening of vibrations, rather as a result of unintended concomitant isometric exercise. Many studies show that isometric contractions increase muscle strength, often to the same extent as dynamic contractions [62] which could explain the greater improvement with the LK position compared to FK for leg strength outcomes in our study. Such isometric contractions of leg muscles would not have influenced upper body strength, and this is supported by the similar strength changes observed for the LK and FK groups in the upper body. Further studies using electromyography (EMG) analysis to measure muscle activation of the upper and lower body during WBV, as well as accelerometer recordings over various body segments to measure vibration transmission with both locked knees and flexed knees posture may assist in determining the effect of stance on muscle contraction and transmission, and thereby refine the WBV prescription to include optimal stance to facilitate vibration to muscle.

### Muscle Morphology

While there were no significant improvements in muscle cross sectional area of the upper or lower limbs, the FK group showed potentially clinically relevant muscle increases at the mid calf compared to the CON group. The lack of significance is likely partially due to a type II error given that our pilot study was inadequately powered for the ES of 0.28 noted in this secondary outcome. Calf muscle strength and size has been associated with gait and balance in older adults [63], and is therefore a potentially important clinical outcome. There are no other studies to our knowledge that have examined changes in muscle cross sectional area using pQCT after WBV exposure in older adults. The need for more robust, long term and sufficiently powered studies is emphasised by our findings.

There are several limitations in our study design. The study was underpowered for the secondary outcomes. Three months of WBV exposure may not have been sufficient to stimulate any musculoskeletal adaptations, particularly in muscle morphology. A four group fully-factorial design would be the optimal design to test the interaction of vibration and knee position on study outcomes. A lack of familiarisation tests prior to baseline assessment, as well as different assessors for baseline and three month testing may have led to results confounded by learning effects and differences due to assessor encouragement and testing experience. We have only tested one vibration magnitude (0.3*g*), the dose recommended by Rubin *et al *[44] to be beneficial to bone and not destructive to osteoblasts. It is possible that a different vibration magnitude is needed for optimal muscle adaptations, and in fact, many other studies have used higher g forces with success for such outcomes [2,32,33]. Specific dose-response studies varying vibration magnitude and total dose are required to refine this aspect of the prescription.

## Conclusions

The results of this pilot RCT have provided some support for the efficacy of WBV exposure as a potential alternative to existing exercise modalities in increasing upper and lower body muscle strength and upper body contraction velocity in healthy older adults. With the aging population leading to a rise in the incidence of musculoskeletal disorders, the efficacy of WBV as a treatment modality warrants further investigation, particularly in mobility-limited cohorts, to assess whether WBV exposure can alleviate muscle wasting due to disease and inactivity. Following the novel and significant changes to upper body muscle function reported, future WBV research should include additional measures of upper body muscle function and morphology to determine the accuracy and reproducibility of our findings. Most importantly, future research must also establish whether improvements from WBV are retained once the vibration stimulus is withdrawn, and whether any meaningful clinical benefits ensue.

## Competing interests

The authors declare that they have no competing interests.

## Authors' contributions

MM and FA carried out the recruitment, baseline testing, training and data entry, MM performed the statistical analyses and drafted the manuscript, FA performed the pQCT scans and analyses, RO and MFS conceived of the study, participated in its design, co-ordination, recruitment and statistical analyses, and revised the manuscript, DG taught the pQCT technique and analysed the pQCT scans. All authors read and approved the manuscript.

## Pre-publication history

The pre-publication history for this paper can be accessed here:

http://www.biomedcentral.com/1471-2318/10/74/prepub

## References

[B1] Bautmans I Van Hees E Lemper JC Mets T Bautmans I Van Hees E Lemper JC Mets T The feasibility of Whole Body Vibration in institutionalised elderly persons and its influence on muscle performance, balance and mobility: a randomised controlled trial [ISRCTN62535013] BMC Geriatr 2005 5 17 10.1186/1471-2318-5-17 16372905 PMC1368976

[B2] Rees SS Murphy AJ Watsford ML Effects of whole-body vibration exercise on lower-extremity muscle strength and power in an older population: a randomized clinical trial Phys Ther 2008 88 4 462 470 10.2522/ptj.20070027 18218826

[B3] Frontera WR Hughes VA Lutz KJ Evans WJ A cross-sectional study of muscle strength and mass in 45- to 78-yr-old men and women J Appl Physiol 1991 71 2 644 650 1938738 10.1152/jappl.1991.71.2.644

[B4] Reed RL Pearlmutter L Yochum K Meredith KE Mooradian AD The relationship between muscle mass and muscle strength in the elderly. [see comment] J Am Geriatr Soc 1991 39 6 555 561 1805811 10.1111/j.1532-5415.1991.tb03592.x

[B5] Fjeldstad C Palmer IJ Bemben MG Bemben DA Whole-body vibration augments resistance training effects on body composition in postmenopausal women Maturitas 2009 63 1 79 83 10.1016/j.maturitas.2009.03.013 19386449

[B6] Baumgartner RN Stauber PM McHugh D Koehler KM Garry PJ Cross-sectional age differences in body composition in persons 60+ years of age J Gerontol A Biol Sci Med Sci 1995 50 6 M307 316 7583802 10.1093/gerona/50a.6.m307

[B7] Going S Williams D Lohman T Aging and body composition: biological changes and methodological issues Exerc Sport Sci Rev 1995 23 411 458 10.1249/00003677-199500230-00016 7556359

[B8] Roubenoff R Hughes VA Sarcopenia: current concepts J Gerontol A Biol Sci Med Sci 2000 55 12 M716 724 11129393 10.1093/gerona/55.12.m716

[B9] Kirkendall DT Garrett WE Jr The effects of aging and training on skeletal muscle Am J Sports Med 1998 26 4 598 602 9689386 10.1177/03635465980260042401

[B10] Taaffe DR Marcus R Musculoskeletal health and the older adult J Rehabil Res Dev 2000 37 2 245 254 10850831

[B11] Tinetti ME Speechley M Ginter SF Risk factors for falls among elderly persons living in the community N Eng J Med 1988 319 26 1701 1707 10.1056/NEJM198812293192604 3205267

[B12] Wolfson L Judge J Whipple R King M Strength is a major factor in balance, gait, and the occurrence of falls J Gerontol A Biol Sci Med Sci 1995 50 Spec No 64 67 7493221 10.1093/gerona/50a.special_issue.64

[B13] Baumgartner RN Khoeler KM Gallagher D Romero L Heymsfield SB Ross RR Garry PJ Lendeman RD Epidemiology of sarcopenia among the elderly in New Mexico J Epidemiol 1998 147 755 763 10.1093/oxfordjournals.aje.a009520 9554417

[B14] Bakhshi V Elliott M Gentili A Godschalk M Mulligan T Testosterone improves rehabilitation outcomes in ill older men. [see comment] J Am Geriatr Soc 2000 48 5 550 553 10811549 10.1111/j.1532-5415.2000.tb05002.x

[B15] Urban RJ Bodenburg YH Gilkison C Foxworth J Coggan AR Wolfe RR Ferrando A Testosterone administration to elderly men increases skeletal muscle strength and protein synthesis Am J Physiol 1995 269 5 Pt 1 E820 826 7491931 10.1152/ajpendo.1995.269.5.E820

[B16] Kenny AM Prestwood KM Gruman CA Marcello KM Raisz LG Effects of transdermal testosterone on bone and muscle in older men with low bioavailable testosterone levels.[see comment] J Gerontol A Biol Sci Med Sci 2001 56 5 M266 272 11320105 10.1093/gerona/56.5.m266

[B17] Snyder PJ Peachey H Hannoush P Berlin JA Loh L Lenrow DA Holmes JH Dlewati A Santanna J Rosen CJ Effect of testosterone treatment on body composition and muscle strength in men over 65 years of age J Clin Endocrinol Metab 1999 84 8 2647 2653 10.1210/jc.84.8.2647 10443654

[B18] Sih R Morley JE Kaiser FE Perry HM Patrick P Ross C Testosterone replacement in older hypogonadal men: a 12-month randomized controlled trial.[see comment] J Clin Endocrinol Metab 1997 82 6 1661 1667 10.1210/jc.82.6.1661 9177359

[B19] Ferrando AA Sheffield-Moore M Yeckel CW Gilkison C Jiang J Achacosa A Lieberman SA Tipton K Wolfe RR Urban RJ Testosterone administration to older men improves muscle function: molecular and physiological mechanisms Am J Physiol Endocrinol Metab 2002 282 3 E601 607 11832363 10.1152/ajpendo.00362.2001

[B20] Balagopal P Schimke JC Ades P Adey D Nair KS Age effect on transcript levels and synthesis rate of muscle MH C and response to resistance exercise Am J. Physiol Endocrinol Metab 2001 280 2 E203 208 11158921 10.1152/ajpendo.2001.280.2.E203

[B21] Fiatarone MA O'Neill EF Ryan ND Clements KM Solares GR Nelson ME Roberts SB Kehayias JJ Lipsitz LA Evans WJ Exercise training and nutritional supplementation for physical frailty in very elderly people. [see comment] N Eng J Med 1994 330 25 1769 1775 10.1056/NEJM199406233302501 8190152

[B22] Borst SE Borst SE Interventions for sarcopenia and muscle weakness in older people. [see comment] Age & Ageing 2004 33 6 548 555 10.1093/ageing/afh201 15385272

[B23] Papadakis MA Grady D Black D Tierney MJ Gooding GA Schambelan M Grunfeld C Growth hormone replacement in healthy older men improves body composition but not functional ability.[see comment] Ann Intern Med 1996 124 8 708 716 8633830 10.7326/0003-4819-124-8-199604150-00002

[B24] Schroeder ET Terk M Sattler FR Androgen therapy improves muscle mass and strength but not muscle quality: results from two studies Am J Physiol Endocrinol Metab 2003 285 1 E16 24 12637255 10.1152/ajpendo.00032.2003

[B25] Verschueren SMP Roelants M Delecluse C Swinnen S Vanderschueren D Boonen S Effect of 6-month whole body vibration training on hip density, muscle strength, and postural control in postmenopausal women: a randomized controlled pilot study J Bone Miner Res 2004 19 3 352 359 10.1359/JBMR.0301245 15040822

[B26] Delecluse C Roelants M Verschueren S Strength increase after whole-body vibration compared with resistance training Med Sci Sport Exerc 2003 35 6 1033 1041 10.1249/01.MSS.0000069752.96438.B0 12783053

[B27] Cardinale M Bosco C The use of vibration as an exercise intervention Exerc Sport Sci Rev 2003 31 1 3 7 10.1097/00003677-200301000-00002 12562163

[B28] Roelants M Delecluse C Verschueren SM Whole-body-vibration training increases knee-extension strength and speed of movement in older women J Am Geriatr Soc 2004 52 6 901 908 10.1111/j.1532-5415.2004.52256.x 15161453

[B29] Rehn B Lidstrom J Skoglund J Lindstrom B Effects on leg muscular performance from whole-body vibration exercise: a systematic review Scand J Med Sci Sports 2007 17 1 2 11 16903900 10.1111/j.1600-0838.2006.00578.x

[B30] Torvinen S Kannus P Sievanen H Jarvinen TAH Pasanen M Kontulainen S Jarvinen TLN Jarvinen M Oja P Vuori I Effect of four-month vertical whole body vibration on performance and balance Med Sci Sport Exerc 2002 34 9 1523 1528 10.1097/00005768-200209000-00020 12218749

[B31] Mahieu NN Witvrouw E Van de Voorde D Michilsens D Arbyn V Van den Broecke W Improving strength and postural control in young skiers: whole-body vibration versus equivalent resistance training J Athlet Train 2006 41 3 286 293 PMC1569559 17043697

[B32] Torvinen S Kannu P Sievanen H Jarvinen TAH Pasanen M Kontulainen S Jarvine TLN Jarvinen M Oja P Vuori I Effect of a vibration exposure on muscular performance and body balance. Randomized cross-over study Clin Physiol Funct Imaging 2002 22 2 145 152 10.1046/j.1365-2281.2002.00410.x 12005157

[B33] Russo C Lauretani F Bandinelli S Bartali B Cavazzini C Guralnik J Ferrucci L High-frequency vibration training increases muscle power in postmenopausal women Arch Phys Med Rehabil 2003 84 12 1854 1857 10.1016/S0003-9993(03)00357-5 14669194

[B34] Rees S Murphy A Watsford M Effects of vibration exercise on muscle performance and mobility in an older population J Aging Phys Act 2007 15 4 367 381 18048942 10.1123/japa.15.4.367

[B35] Fagnani F Giombini A Di CA Pigozzi F Di SV The effects of a whole-body vibration program on muscle performance and flexibility in female athletes Am J Phys Med Rehabil 2006 85 12 956 962 10.1097/01.phm.0000247652.94486.92 17117001

[B36] Kawanabe K Kawashima A Sashimoto I Takeda T Sato Y Iwamoto J Effect of whole-body vibration exercise and muscle strengthening, balance, and walking exercises on walking ability in the elderly Keio J Med 2007 56 1 28 33 10.2302/kjm.56.28 17392595

[B37] Cheung W Mok H Qin L Sze P Lee K Leung K High-frequency whole-body vibration improves balancing ability in elderly women Arch Phys Med Rehabi 2007 88 7 852 857 10.1016/j.apmr.2007.03.028 17601464

[B38] Bruyere O Wuidart MA Di Palma E Gourlay M Ethgen O Richy F Reginster JY Controlled whole body vibration to decrease fall risk and improve health-related quality of life of nursing home residents Arch Phys Med Rehabil 2005 86 2 303 307 10.1016/j.apmr.2004.05.019 15706558

[B39] Ahlborg L Andersson C Julin P Whole-body vibration training compared with resistance training: effect on spasticity, muscle strength and motor performance in adults with cerebral palsy J Rehabil Med 2006 38 5 302 308 10.1080/16501970600680262 16931460

[B40] Turbanski S Haas CT Schmidtbleicher D Friedrich A Duisberg P Turbanski S Haas CT Schmidtbleicher D Friedrich A Duisberg P Effects of random whole-body vibration on postural control in Parkinson's disease Res Sports Med 2005 13 3 243 256 16392539 10.1080/15438620500222588

[B41] Roelants M Delecluse C Goris M Verschueren S Effects of 24 weeks of whole body vibration training on body composition and muscle strength in untrained females Int J Sports Med 2004 25 1 1 5 10.1055/s-2003-45238 14750005

[B42] Rubin C Recker R Cullen D Ryaby J McCabe J McLeod K Prevention of postmenopausal bone loss by a low-magnitude, high-frequency mechanical stimuli: a clinical trial assessing compliance, efficacy, and safety J Bone Miner Res 2004 19 3 343 351 10.1359/JBMR.0301251 15040821

[B43] Jordan MJ "Vibration Training: an Overview of the Area, Training Consequences, and future considerations" J Strength Cond Res 2005 19 2 459 466 10.1519/13293.1 15903391

[B44] Rubin C Pope M Fritton JC Magnusson M Hansson T McLeod K Transmissibility of 15-hertz to 35-hertz vibrations to the human hip and lumbar spine: determining the physiologic feasibility of delivering low-level anabolic mechanical stimuli to skeletal regions at greatest risk of fracture because of osteoporosis Spine 2003 28 23 2621 2627 10.1097/01.BRS.0000102682.61791.C9 14652479

[B45] Hazell T Jakobi J Kenno K The effects of whole-body vibration on upper- and lower-body EMG during static and dynamic contractions Appl Physiol Nutr Metab 2007 32 6 1156 63 10.1139/H07-116 18059590

[B46] Roelants M Verschueren SMP Delecluse C Levin O Stijnen V Whole-body-vibration-induced increase in leg muscle activity during different squat exercises J Strength Cond Res 2006 20 1 124 9 10.1519/R-16674.1 16503671

[B47] Dallal GE 2009 http://www.randomization.com

[B48] Kiiski J Heinonen A Jaervinen TL Kannus P Sievanen H Transmission of vertical whole body vibration to the human body J Bone Miner Res 2008 23 8 1318 1325 10.1359/jbmr.080315 18348698

[B49] Orr R de Vos NJ Singh NA Ross DA Stavrinos TM Singh Fiatarone MA Power Training Improves Balance in Healthy Older Adults J Gerontol A Biol Sci Med Sci 2006 61A 1 78 85 10.1093/gerona/61.1.78 16456197

[B50] Kervio G Carre F Ville NS Kervio G Carre F Ville NS Reliability and intensity of the six-minute walk test in healthy elderly subjects Med Sci Sport Exerc 2003 35 1 169 174 10.1097/00005768-200301000-00025 12544651

[B51] Washburn RSK Jetter A Janney C The physical activity scale for the elderly (PASE): development and evaluation J Clin Epidemiol 1993 46 153 162 10.1016/0895-4356(93)90053-4 8437031

[B52] Mayr S Errdfelder E Buchner A Faul F A short tutorial of G Power Tutorials Quant Method Psychol 2007 3 51 59

[B53] Dawson-Hughes B Harris S Regional changes in body composition by time of year in healthy postmenopausal women Am J Clin Nutr 1992 56 2 307 313 1636609 10.1093/ajcn/56.2.307

[B54] Effect Size Calculator: a user guide to using the spreadsheet http://www.pipsproject.org/RenderPagePrint.asp?LinkID=30325017

[B55] Coe R It's the Effect Size, Stupid British Educational Research Association Annual Conference, Exeter 2002

[B56] de Vos NJ Singh NA Ross DA Stavrinos TM Orr R Fiatarone Singh MA Optimal load for increasing muscle power during explosive resistance training in older adults J Gerontol A Biol Sci Med Sci 2005 60 5 638 647 15972618 10.1093/gerona/60.5.638

[B57] Metter EJ Conwit R Tobin J Fozard J Age-Associated Loss of Power and Strength in the Upper Extremities in Women and Men J Gerontol A Biol Sci Med Sci 1997 52 B2 67 76 10.1093/gerona/52a.5.b267 9310077

[B58] Skelton DA Greig CA Davies JM Young A Strength, power and related functional ability of healthy people aged 65-89 years Age & Ageing 1994 23 5 371 377 10.1093/ageing/23.5.371 7825481

[B59] Bassey EJ Fiatarone MA O'Neill EF Kelly M Evans WJ Lipsitz LA Leg extensor power and functional performance in very old men and women Clin Sci 1992 82 3 321 327 1312417 10.1042/cs0820321

[B60] de Vos NJ Singh NA Ross D Stavrinos TM Orr R Fiatarone Singh MA Effect of Explosive Resistance Training Intensity on the Contribution of Force and Velocity to Peak Power Generation in Older Adults: A Randomized Controlled Trial J Aging & Phys Act 2008 16 4 393 407 10.1123/japa.16.4.393 19033601

[B61] Salem GJ Skinner JS Chodzko-Zajko WJ Proctor DN Fiatarone Singh MA Minson CT Nigg CR Exercise and Physical Activity for Older Adults Med Sci Sport Exerc 2009 41 7 1510 1530 10.1249/MSS.0b013e3181a0c95c 19516148

[B62] Symons TB Vandervoort AA Rice CL Overend TJ Marsh GD Symons TB Vandervoort AA Rice CL Overend TJ Marsh GD Effects of maximal isometric and isokinetic resistance training on strength and functional mobility in older adults J Gerontol A Biol Sci Med Sci 2005 60 6 777 781 15983182 10.1093/gerona/60.6.777

[B63] Chandler JM Duncan PW Kochersberger G Studenski S Is lower extremity strength gain associated with improvement in physical performance and disability in frail community-dwelling elders? Arch Phys Med Rehabil 1998 79 1 24 30 10.1016/S0003-9993(98)90202-7 9440412

